# Chemosensory Event-Related Potentials and Power Spectrum Could Be a Possible Biomarker in 3M Syndrome Infants?

**DOI:** 10.3390/brainsci10040201

**Published:** 2020-03-30

**Authors:** Sara Invitto, Alberto Grasso, Dario Domenico Lofrumento, Vincenzo Ciccarese, Angela Paladini, Pasquale Paladini, Raffaella Marulli, Vilfredo De Pascalis, Matteo Polsinelli, Giuseppe Placidi

**Affiliations:** 1 Laboratory of Cognitive and Psychophysiological Olfactory Processes, Department of Biological and Environmental Sciences and Technologies, University of Salento, 73100 Lecce, Italy; grassoalberto42@yahoo.it; 2 Human Anatomy and Neuroscience Lab, Department of Biological and Environmental Science and Technologies, University of Salento, 73100 Lecce, Italy; dario.lofrumento@unisalento.it; 3 Santa Chiara Institute, 00144 Roma, Italy; ciccareseenzo@libero.it; 4 Neonatology Unit, Policlinico Universitario Gemelli, Università Cattolica del Sacro Cuore, 00168 Roma, Italy; angelapaladini17@gmail.com; 5 Pediatric Unit, Vito Fazzi Hospital, 73100 Lecce, Italy; linopaladini@alice.it; 6 Neurology Unit, Vito Fazzi Hospital, 73100 Lecce, Italy; raffae83@libero.it; 7 Department of Psychology, University ‘La Sapienza’, 00185 Roma, Italy; vilfredo.depascalis@uniroma1.it; 8 A2VI-Lab c/o Department of Life, Health & Environmental Sciences, University of L’Aquila, 67100 L’Aquila, Italy; matteo.polsinelli@graduate.univaq.it (M.P.); giuseppe.placidi@univaq.it (G.P.)

**Keywords:** CSERP, OERP, EEG, spectra power, olfactory system, 3M syndrome, rare disease

## Abstract

3M syndrome is a rare disorder that involves the gene cullin-7 (*CUL7)*. CUL7 modulates odour detection, conditions the olfactory response (OR) and plays a role in the development of the olfactory system. Despite this involvement, there are no direct studies on olfactory functional effects in 3M syndrome. The purpose of the present work was to analyse the cortical OR through chemosensory event-related potentials (CSERPs) and power spectra calculated by electroencephalogram (EEG) signals recorded in 3M infants: two twins (3M-N) and an additional subject (3M-O). The results suggest that olfactory processing is diversified. Comparison of N1 and Late Positive Component (LPC) indicated substantial differences in 3M syndrome that may be a consequence of a modified olfactory processing pattern. Moreover, the presence of delta rhythms in 3M-O and 3M-N clearly indicates their involvement with OR, since the delta rhythm is closely connected to chemosensory perception, in particular to olfactory perception.

## 1. Introduction

3M syndrome is a “rare autosomal recessive dwarf syndrome” [1]. The distinctive features of this little-known syndrome are limited prenatal growth, facial dysmorphism, absence of microcephaly and cognitive impairment [2]. Since 3M syndrome is autosomal recessive, both inherited copies of the gene have mutations. Mutually exclusive genetic mutations in cullin-7 (*CUL7*), obscurin-like 1 (*OBSL1*) and coiled-coil domain-containing protein 8 (*CCDC8*) cause the pathology, as confirmed by a study conducted by Dan Hanson and collaborators [3]. They noted that, in terms of the clinical and biochemical 3M syndrome phenotype, children with *CUL7* mutations were significantly shorter than those with *OBSL1* or *CCDC8* mutations. However, the aetiological mechanisms that lead to the observed growth disability in 3M syndrome remain unclear, but they are probably related to abnormalities in basic cell growth and changes in cellular responses to growth factor stimulation. 

Although 3M syndrome is considered a relatively rare disease, it is probably an under-recognised condition; its main characteristics, including impaired pre- and post-natal growth, are shared with all gestational age children with growth failure. This population includes many children who do not yet have a clear mechanism of growth impairment [4]. It is likely that 3M syndrome is often misdiagnosed or unrecognised due to normal mental development, mild dysmorphic facial features and good patient health. 

Residual clinical features (triangular face, pointed chin, mouth and prominent lips, fleshy nose with anteverted nostrils, short stature, large skull and prominent forehead) and clinical history (low birth weight) are typical of 3M syndrome [1]. Epidemiological data about 3M syndrome are not known. Today, approximately 200 cases have been reported worldwide [5]. 

### 1.1. CUL7 Mutation and Potential Olfactory Involvement

As mentioned above, genetically confirmed patients with 3M syndrome carry mutations in *CCDC8* (5%), *OBSL1* (25%) or *CUL7* (70%) [6]. CUL7 interacts with other cellular proteins and contributes to the formation of an E3 ubiquitin ligase complex that ubiquitinates specific targets. *CUL7* mutations may disrupt insulin-like growth factor 1 (IGF-1) and growth hormone (GH) signalling pathways and contribute to growth alteration [5]. Insulin receptor substrate 1 (IRS1) is a target of the CUL7-SCF ubiquitin ligase. IRS1 is a signalling molecule that is a member of a family of adaptor molecules downstream of GH, IGF-1 and insulin receptors [6,7]. Insulin receptors are expressed in olfactory receptor neurones of rat olfactory mucosa, a fact that suggest insulin plays a role in odour detection modulation at the olfactory mucosa level [8,9]. 

CUL7-FBXW8 is a component of an E3 ubiquitin ligase that localises to the Golgi apparatus in neurones and is required for dendrite growth and organisation. Inhibition of this ligase in neurones alters Golgi morphology, impairs vesicle trafficking and disrupts dendrite morphogenesis and arborisation [10]. The ubiquitin ligase activity is linked to axon guidance during pathfinding in the development of the olfactory system.

### 1.2. Olfactory Perception and Chemosensory Event-Related Potentials (CSERPs) in Infants

Olfactory perception is highly developed in newborns and infants. Recent research indicates that olfactory system activity is already present in 1-day-old newborns [11,12]. Furthermore, smells can modulate nociception [13], by inducing greater stability during painful procedures and lower severity of central apnoea. Moreover, unpleasant or irritating odours promote disadvantageous evolutionary responses, such as decreased respiratory rate (up to apnoea) [14]. The sense of smell is also compromised in children with cerebral malformations, genetic diseases (e.g., trisomy 13 or 18, Kallmann syndrome or Riley-Day syndrome), endocrine disorders (such as hypothyroidism and gonadal dysgeneses) and in infants borne to diabetic mothers [15]. 

A recent work showed that it is possible to record olfactory event-related potentials (OERPs) in infants [16]. OERPs and CSERPs are electrophysiological components that allow researchers to evaluate chemosensory and chemoperceptual responses to olfactory stimuli [17]. The main difference between OERP and CSERPs is that the former is elicited by purely olfactory stimulation, while the latter is elicited by chemical stimulation, which may also include trigeminal activation [18].

Schriever and colleagues research, however, highlights the difficulty in observing OERPs in infants. This phenomenon is likely because there are more recording artefacts. OERP components in infants are the same as in adults: early components N1 and P2 [16] and late positive components (LPCs) [19]. N1 and P2 are the early sensorial components and are modulated by stimulus concentration and typology. LPCs include P3a and P3b and are modulated by the cognitive aspects of the stimulus (e.g., presentation frequency or stimulus salience) [19]. Moreover, time-frequency analysis highlights increased low frequencies (4–7 Hz) in a temporal range that corresponds to LPC [16]. 

Even though there are no previous ERP (and specifically CSERPs) studies in 3M infants, one could hypothesise that the olfactory system could be dysfunctional in infants with *CUL7* mutations [20]. Based on the integrated CSERPs approach, the aim of this study was to investigate whether there are implications at the level of olfactory perception both in OERP components and with regard to the main rhythms associated with rhinoencephalon [21] and entorhinal cortex [22] activity in 3M syndrome. Since no study has evaluated the use of CSERPs or OERPs to investigate olfactory functional responses in 3M infants, olfactory function in this rare syndrome is poorly characterised. Moreover, to the best of our knowledge, no study has been conducted using electroencephalogram (EEG) signals from the subjects with 3M syndrome using signal processing and analysis strategies.

There are multiple potential benefits from this study. If the 3M syndrome subjects differ from the controls with respect to the olfactory response, early OERP screening, which represents an economic and non-invasive tool compared to genetic screening, could then lead to a possible subsequent genetic investigation (if it is positive). Furthermore, this research could allow us to deduce functional *CUL7* involvement in the human chemosensory/olfactory response, a prospect that has not yet been studied.

## 2. Materials and Methods

The research was conducted at the Neurology Unit of the Vito Fazzi Hospital in Lecce with subjects recruited at the Neonatal Intensive Care Unit (UTIN). Data collection was performed in compliance with the Code of Ethics of the World Medical Association (Declaration of Helsinki) and authorized by the ASL-Lecce Ethics Committee (Approval record N°7, Date 19 July 2017). Written informed consent was obtained from the parents.

### 2.1. Subjects

Three subjects (males) with a diagnosis of 3M syndrome were recruited for the study. The subjects were siblings, two 5-month-old twins (3M-N) and their 18-month-old brother (3M-O). *CUL7* genetic analysis (exons 14-23/24) highlighted the presence of pathogenic variants c2781delC (p.Ser928Leufs*5) and c.4391 A>C (p.His1464Pro) in the state of compound heterozygosity. The diagnostic conclusion for all three siblings was 3M syndrome due to familiar mutations. The laboratory data is compatible with the segregation of the family pathology in the foetus. The 3M syndrome group presented the following medical history: prematurity, low birth weight (LBW), small size for gestational age, syndromic facies, triangular face, prominent frontal drafts, bulbous nose, flat angiomas of the median line, short neck and thorax, hypospadias and suspected bow curvature, fleshy and prominent heels, prenatal 3M diagnosis based on amniocentesis karyotype, glandular hypospadias, transient hypocalcaemia and transient oliguria. Moreover, the subjects with 3M syndrome showed a larger cranial circumference (75–90°). Our sample size represents about 1.5% of the approximately 200 cases described worldwide [5].

The control group was recruited with the criterion of having the same gestational and post-conceptional ages as the 3M subjects, but no apparent clinical abnormalities from anamnestic data. The controls consisted of two healthy 12-month-old male twins (HS-O) and two 4-month-old male twins (HS-N). 

The subjects were compared, separately, according the post conceptional age; the HS-O twin pair served as controls for the 3M-O and the two HS-N served as controls for the 3M-N. Independent comparisons were performed, due to different characteristics from neonatal EEGs and developmental brain behaviour [23]. Additionally, all infants were examined by the hospital paediatrician to rule out nasal congestion or other temporary respiratory diseases. Both healthy and subjects with 3M syndrome were subjected to neonatal auditory screening and the auditory brainstem response (ABR) and did not show any significant clinical abnormalities. No other behavioural olfactory or chemosensory assessment was performed.

### 2.2. OERP Assessment

Subjects performed a CSERPs task that involved the eucalyptus scent (natural eucalyptol oil, 1,3,3-Trimethyl-2-oxabicyclo [2.2.2] octane; Sigma-Aldrich, CAS Number 470-82-6). The experimental eucalyptus concentration was 20 μL in 10 mL Vaseline oil. The odorous solutions were prepared in 20 mL transparent glass vials and kept sealed with plastic film in a darkened cabinet. The scent was administered via an olfactometer [24]. 

The OERP presentation paradigm consisted of sequences of olfactory stimulations; each stimulation lasted 340 ms, with an inter-stimulus interval (ISI) of 20 s. In total, the subjects were exposed to 20 stimulations (a sufficient number of stimulations, because the minimum number to elicit OERP is 8 [16]). In accordance with recommendations based on previous research, the ISI was greater than 10 s to avoid habituation [20]. The device used to record the presentation of odorous stimuli allowed us to measure, in a controlled and automated way, the CSERPs evoked by olfactory stimuli synchronized to the acquisition of the EEG signal. The administration of the odorant, which took place through the olfactometer, was presented through a plexiglass tube that was positioned in the centre of the two nostrils. The odorant was delivered as binarinal stimuli in front of the nose. During the electroencephalographic recording, the children were seated in the arms of the mother, who in turn was sitting in a comfortable armchair placed inside the EEG recording room. The children were in a relaxed condition, were in a post-prandial state (i.e., they had eaten about an hour before the EEG recording) [25] and were in a waking state [26]. The choice of the eucalyptol odorant, which has a mixed component both olfactory and trigeminal, allowed us to keep the children in arousal during the CSERPs recordings [27,28,29,30].

### 2.3. EEG Recording

The EEG signals were recorded using a Micromed 19-channel amplifier (Fp1; Fp2; F7; F3; Fz; F4; F8; T3; C3; Cz; C4; T4; T5; P3; Pz; P4; T6; O1; and O2). The scalp electrodes were applied according to the International 10-20 system. The EEG signal processing was performed using a Brain Vision Analyzer (Brain Products GmbH). The impedance was maintained below 8 kΩ, and the sampling rate was 256 Hz.

### 2.4. Data Processing

#### 2.4.1. OERP Pre-Processing

The electrodes were online referenced to FCz, and offline, they were postponed with a common offline reference [31]. The signal was filtered offline (0.01–50 Hz, 24 dB), and the artefact rejection threshold was set to > 125 [32]. ERP epochs included a 100-ms pre-stimulus reference period and a 500-ms post-stimulus segment. The peaks were automatically detected for all channels. The OERP components were labelled as N1 and LPC according to Pause et al. [19]. The latency windows were set to 100–400 ms for N1 and 350–600 ms for LPC [20,33]. Main regions of interest (ROIs) were extracted though the linear derivation process: central left (C3-A1-T3), central right (C4-A2-T4), temporo-parietal left (P3-T5-O1), temporo-parietal right, (P4-T6-O2), frontal left (Fp1-F3-F7), frontal right (Fp2-F4-F8), central (Cz), parietal (Pz) and frontal (Fz). This process was defined *a priori* to reduce the number of electrode/channel comparisons, according to the definition of the two hemispheres and the lobes [34]. The linear derivation process allows one to synthetize new channels from linear combinations of recording existing electrodes/channels [35].

#### 2.4.2. EEG Signal Pre-Processing

We further analysed the original EEG signals with signal processing strategies, since the sample was necessarily small and the study could be reduced exclusively to a single case. Thus, we investigated EEG rhythms on pieces (trials) of signal collected after each olfactory stimulation by searching for the presence of recurrent common trends in the subjects with 3M syndrome with respect to the controls. 

Signals were filtered with an offline modality by considering only 1-s trials, starting with the onset of the olfactory stimulation, since the brain response exhibits a decaying signal that has a zero value 1 s after olfactory stimulation. Ocular rejection was performed by independent component analysis (ICA). First, to correct different amplification effects, each trial was normalised with respect to its baseline level, obtained by calculating the mean value of the power spectrum in a frequency band (65–75 Hz), which is usually only occupied by noise. Thus, all the resulting trials showed the same amplification. Then, the signal was subjected to a band-pass filter (0.01–49 Hz, 24 dB) in the frequency domain in order to eliminate noise and offset. Finally, each trial was elaborated for eliminating artefacts. To this end, independent component analysis (ICA) was performed and the components were calculated. Each component was defined by an array of weights that represented the magnitude of each channel related to that component. For each of the resulting components, the correlation with the corresponding components of two reference channels (A1 and A2) was calculated; the component was eliminated if the correlation was, in modulus, greater than a threshold value t (i.e., t = 0.5). Residual artefacts due to ocular movements (blink and horizontal and vertical movements), local electrical disturbance/interference and heartbeat, were also treated with ICA but in a different manner. Specifically, we used the fact that weights representing each component can be interpolated in 3D (the spatial localisation of the channels on the scalp) and projected on a 2D scalp map, the topoplot [36]. In this way, we obtained a localisation of the component on the head. The shape of the resulting topoplots allowed one to relate each component as an artefact [36,37]. The residual components were projected back to the signal space to obtain a filtered version of the signal that composed each trial.

## 3. Results

### 3.1. OERP Data Analysis

**Figure 1:**
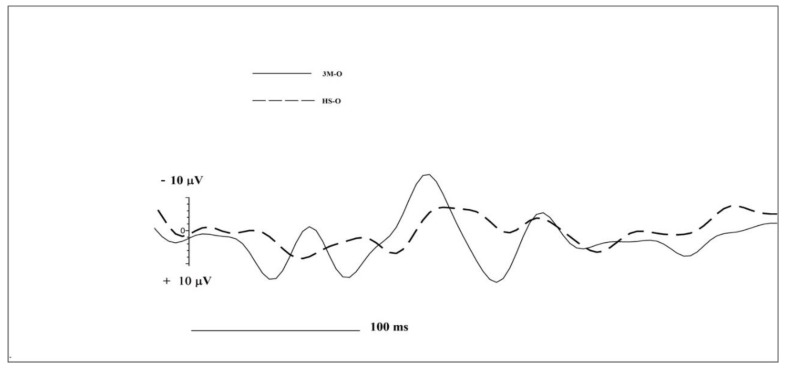
Central left Region of Interest (ROI) comparison of Chemosensory Event Related Potentials (CSERPs) components for 3M-O (black continuous line) and HS-O (dashed line) subjects.

**Figure 2:**
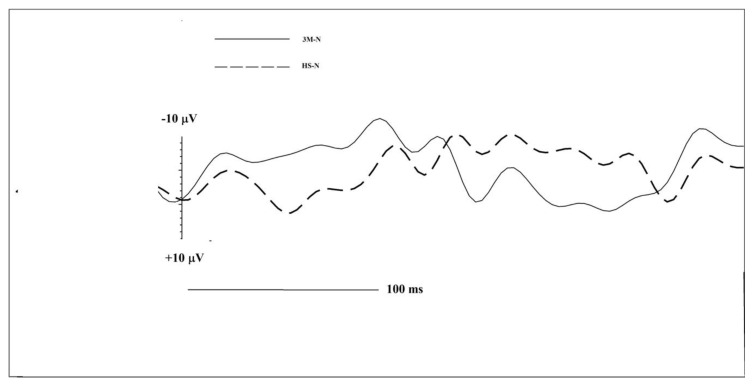
Central left ROI comparison of CSERPs components for 3M-N (black continuous line) and HS-N (dashed line) subjects.

Due to the sample size, we performed initial explorative and descriptive analyses to investigate N1 and LPC OERP components (Table 1 and Table 2). The explorative analysis showed an OERP trend for greater amplitudes in subjects with 3M syndrome, and this trend was apparently more uniform and structured in 3M-O (Figure 1) compared to 3M-N (Figure 2). The OERP results revealed that 3M-O showed greater N1 amplitudes and faster latencies on frontal left (3M-O 17.34 µV vs. HS-O 1.42 µV), frontal right (3M-O −25.37µV vs. HS-O −12.55 µV), central left (3M-O −11.17 µV vs. HS-O −4.1 µV), central right (3M-O −7.97 µV vs. HS-O −2.7 µV) and temporal left (3M-O −6.8 µV vs. HS-O −2.2 µV). Cz showed faster latency (3M-O 109 ms vs. 129 ms) and Pz showed greater amplitude (3M-O −2.80 µV vs. HS-O −1.34 µV) in 3M-O. LPC data followed the same pattern as N1, except for central right (3M-O 9.75 µV vs. HS-O 12.23 µV), Fz (3M-O 5.78 µV vs. HS-O 9.58 µV) and Pz (3M-O 4.26 µV vs. HS-O 22.06 µV), where 3M-O had a decreased amplitude. 

For N1, the 3M-N twins showed increased amplitude in frontal right (3M-N −9.32 µV vs. HS-N −2.38 µV), central left (3M-N −7.68 µV vs. HS-N-5.76 µV), Cz (3M-N −16.02 µV vs. HS-N −11.80 µV) and Fz (3M-N −8.95 µV vs. HS-N −6.67 µV). N1 latencies were faster in the frontal left (3M-N 121 ms vs. HS-N 203 ms), central left (3M-N 121 ms vs. HS-N 168 ms), temporal left (3M-N 297 ms vs. HS-N 316 ms), temporal right (3M-N 207 ms vs. HS-N 270 ms), Fz (3M-N 160 ms vs. HS-N 250 ms) and Pz (3M-N 164 ms vs. HS-N 188 ms) in 3M-N. 

LPC data revealed that 3M-H had a greater amplitude in frontal left (3M-N 6.56 µV vs. HS-N 4.89 µV), central left (3M-N 5.88 µV vs. HS-N 4.19 µV) and Pz (3M-N 14.27 µV vs. HS-N 5.58 µV). There was no identifiable typified LPC [16] for 3M-N and HS-N in frontal right and for 3M-N in Cz. All 3M-N ROIs exhibited shorter latencies compared to HS-N.

### 3.2. EEG Spectral Analysis

**Figure 3:**
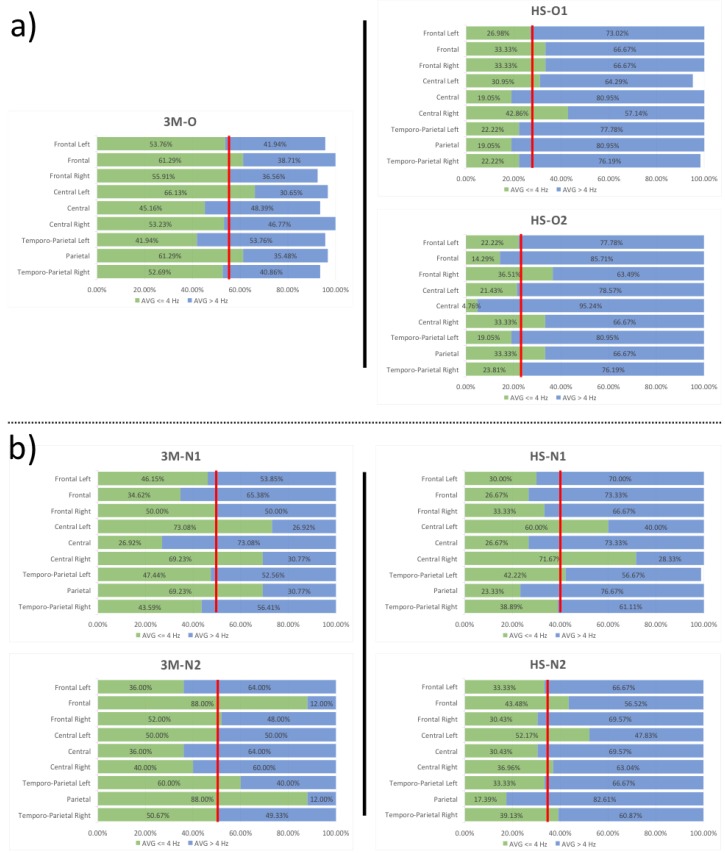
Representation of the percentage of trials (horizontal axis) divided by ROIs (vertical axis), for which 60% of the power spectrum area was ≤ 4 Hz (green) or > 4 Hz (blue) for each of the treated infants. A sum less than 100% indicates that some trials were too corrupted to be treated and, hence, discarded; this phenomenon mainly occurred for subject 3M-O. Data regarding subject 3M-O and the corresponding controls HS-O1/HS-O2 are reported in a) and 3M-N1/3M-N2 and the corresponding shared controls are reported in b). Vertical bars indicate the average threshold; differences are apparent between patients and controls.

After pre-processing, each trial was analysed 0–1000 ms after onset (since the brain response signal is zero 1 s after olfactory stimulation). The resulting signals, each sampled for 1 s at 256 points, were analysed with Fourier transform (FT) in 4 Hz windows (0.01–4, 4–8, 8–12, 12–16, etc., until 48 Hz), and the power spectrum was calculated [38]. The analysis was performed for each trial and each channel separately, and the results were analysed in the form of a power spectrum represented graphically and as topoplot images. The obtained results demonstrated that the frequencies generated by olfactory stimulations mostly occurred in the (0.01,8] Hz interval in both the subjects with 3M syndrome and healthy subjects. However, the examined subjects with 3M syndrome had low frequencies (≤ 4 Hz) elicited by olfactory stimulation, while higher frequencies (> 4 Hz) were mostly activated for healthy subjects. Figure 3 highlights these aspects by reporting, for all subjects and for each channel, the percentage of trials for which 60% of the EEG power spectrum area was in the (0.01, 4] Hz interval (green) or > 4 Hz (blue). Vertical red lines indicate the mean percentage (averaged for all ROIs) of 60% of the power that occurred before 4 Hz. This presentation clearly shows a right displacement for patients with 3M syndrome with respect to the corresponding controls. These data confirm, for subjects with 3M syndrome, the increment of trials for which the power spectrum concentrated in the (0.01, 4) Hz interval.

**Figure 4:**
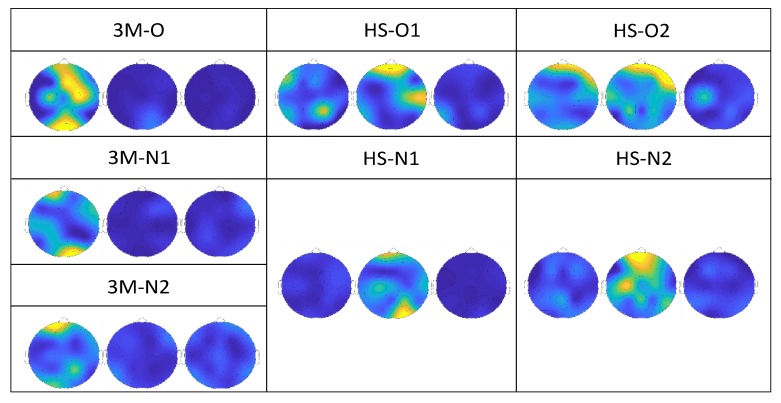
Topoplot images that report the power spectrum distribution of one of the typical trials for each infant. Each of the three topoplots refers to an analysed bandwidth: 0.01–4 Hz (left), 4–8 Hz (middle) and 8–12 Hz (right). The scale was normalized between 0 and 1 (0 = intense blue, 1 = intense yellow) for all subjects and is not shown for convenience. The data disposition resembles that of Figure 1. For patients with 3M syndrome (left column), the left topoplot (0.01–4 Hz) carried most of the power; for healthy subjects (middle and right columns), most of the power was concentrated in the middle topoplot (4–8 Hz). For all subjects, the right topoplot (8–12 Hz) contained negligible power with respect to the lower frequency windows.

This effect was most noticeable between 3M-O (row #1, column #1) and HS-O (row #1, columns #2 and #3) with respect to 3M-N (rows #2 and #3) and HS-N (row #2, columns #2 and #3). Moreover, some ROIs were more involved than others in this process, as shown in Figure 4, which reports the percentage difference of the green area in Figure 3 between patients with 3M syndrome and corresponding controls (and separated by ROIs). Table 3 describes a ROI analytic evaluation showing percentage difference between the green regions (area of the power spectrum ≤ 4 Hz) in Figure 3 for subjects with 3M syndrome and control subjects; Table 3 confirms that, for 3M-O, the power spectrum was concentrated in the (0.01, 4] Hz interval (positive values, highlighted in “orange”) for all ROIs with respect to both controls. However, for 3M-N, this behaviour was confirmed just for some specific ROIs (regions with discordant signs were not considered).

We observed larger differences in EEG spectral power displacement in the subjects with 3M syndrome, although this effect was different between 3M-O and 3M-N. In particular, 3M-O showed more activation in all the ROIs, but 3M-N showed more activation in temporo-parietal (right and left), parietal, frontal right and frontal left, where both clinical samples exhibited intersection. These results highlight that similar EEG patterns are present in the same clinical categorisation (e.g., 3M). The signal behaviour between 3M-O and its corresponding control is differentially distributed with respect to 3M-N and HS-N, although the general effect was the same. 3M-N had very similar responses; the different responses between patients of different ages were more pronounced than those between controls of different ages, where similar patterns, although at different scales, were maintained. 

Figure 4 shows the screenshots of typical power spectrum topoplots (with normalised scales) in the first three frequency windows (0.01–4 Hz, 4–8 Hz and 8–12 Hz, respectively) for one trial for each of the analysed subjects. Notably, Figure 4 confirms that patients with 3M syndrome responded more in the 0.01–4 Hz bandwidth, while healthy subjects were mostly active in the 4–8 Hz bandwidth. Power was negligible in the 8–12 Hz bandwidth for all subjects. 

## 4. Discussion and Conclusions

3M syndrome is extremely rare and difficult to diagnose. Its peculiarity lies in bone alterations and genetic variations, which, among various aspects that these changes modulate, also affects the olfactory response. Indeed, *CUL7*, a gene involved in the 3M syndrome, can modulate odour detection and condition the OR and plays a role in the development of the olfactory system [3,9,10,39]. Despite this involvement, there are no direct studies on the functional effects of this syndrome. This paucity of data is due to the fact that the syndrome is one of the rarest genetic disorders and evaluation of cortical responses to olfactory stimuli in infants and newborns is one of the less frequent investigations within psychophysiology and cognitive neuroscience [16,40]. 

The purpose of the present work was to analyse the cortical olfactory response, recorded through CSERPs, in infants with 3M syndrome. We first evaluated the CSERPs responses with a direct descriptive comparison (since this study was comparable to a single case study due to the small sample size) on the trends of the olfactory stimulus sensory and perceptive components. In particular, we analysed the sensory components N1 and LPC elicited by the stimulation paradigm [19]. The CSERPs results demonstrated that the 3M-O infant exhibited increased N1 amplitudes and faster latencies. Furthermore, we found a faster latency in Cz, which is positioned on the precentral gyrus [41], and greater amplitude in Pz, which is located in the middle parietal lobe. The precentral gyrus and parietal cortex are considered sites for olfactory working memory [42]. We interpret these findings as an indication of greater allocation of attentional resources, enhanced olfactory working memory and olfactory perception, which is visible in the N1 component, which is involved in the sensorial detection of olfactory stimulation [19]. We suppose that this enhancement could be related to the *CUL7* alteration. LPC data followed the same results as N1, except for central right, where we observed a decreased amplitude. 

The wider LPC is a consequence of the processing of olfactory information visible through the N1 component. The 3M-N twins also showed increased amplitude in the precentral gyrus and faster N1 and LPC latencies, although the results in younger infants were apparently less defined and exhibited less LPC typing than for the 3M-O and control groups. This minor typing is evident with the difficulty of identifying the LPC in frontal right, central right and Cz ROIs [16].

In 3M syndrome, olfactory processing appears to be clearly diversified. Specifically, comparison of the N1 and LPC indicates substantial differences in 3M syndrome that may be a consequence of a modified olfactory processing pattern. Moreover, the subjects with 3M syndrome showed different arousal localisations from olfactory stimulation, data that implicate much larger areas that range from the left hemisphere to the midline sites (i.e., Fz, Cz and Pz). These differences were more distributed and evident in the infant rather than younger twins, but in general, they seemed to be constant with respect to the CSERPs trend. As a further signal control, we performed a new analysis based on the assumption that the slow and high CSERPs frequencies are related [43]. Indeed, we considered the rhythms within the signal and considered the greater cortical response, which in our case coincided, at a temporal level, with the CSERPs-elicited response. These results demonstrated that the frequencies generated by olfactory stimulations were mostly present in the (0.01,8] Hz interval in subjects with 3M syndrome and healthy subjects. However, the behaviour observed in the examined subjects was that low frequencies, in particular δ (≤ 4 Hz) were elicited by olfactory stimulation in subjects with 3M syndrome, while higher frequencies (> 4 Hz) were mostly activated for healthy subjects. Moreover, we argue that some ROIs are more involved than others in this process. In particular, 3M-O showed involvement in all ROIs, although parietal, central left, central, frontal and frontal left exhibited greater activation; 3M-N showed elevated activation in temporo-parietal (left and right), parietal, frontal left and frontal right. Overall, similar EEG patterns were present for the same clinical categorisation (e.g., 3M-O and 3M-N). The δ EEG rhythm appears to be more structured in 3M-O, although the general effect in 3M-N was the same, but less strong. The different age-related responses in 3M infants were more pronounced than those between controls, where similar patterns were maintained in CSERPs and EEG spectral analysis. Moreover, the presence of δ rhythms in patients with 3M syndrome clearly implicates olfactory response involvement, since this rhythm is closely connected to olfactory perception [44,45]. 

Although we were unable to perform robust statistical analysis due to the limited number of subjects, our results are the first assessing, in a preliminary way, CSERPs in 3M subjects, and this could be of interest for basic research and clinicians. For basic research, these results highlight, for the first time in human infants, a functional aspect of the cortical olfactory response linked to the *CUL7* gene. From the clinical point of view, these results suggest that a diagnostic evaluation of the cortical olfactory response at an early age may provide indications for subsequent genetic screening, which is more complex and expensive than a CSERPs assessment. The first limitation of this preliminary study comes from the sampling of 3M subjects. These subjects, in fact, belong to the same family, therefore they could show similar electrophysiological characteristics due to their familiarity and not due, exclusively, to the olfactory system, despite the peculiarity of these subjects is precisely having a variation of the CUL7 gene, closely connected with olfaction. The other limitations concern the chemical nature of stimulation and the sample size. In fact, regarding the first one, we did not use a purely olfactory stimulus (e.g., phenethyl alcohol) to prevent the child from relaxing and falling asleep during the EEG recording [30]. The administration of eucalyptus, in fact, on the one hand allowed us to keep the children in a state of mood increased vigilance, but on the other hand has ensured that the elicited component is of a mixed type (both olfactory and trigeminal) [18,30,46].

The sample size, even if it is a limitation, also partly represents a strength. The small number of subjects actually represents a larger percentage of subjects with 3M syndrome than the percentage that would usually be represented by afflicted individuals in clinical studies. We can conclude, albeit in a preliminary way, that the chemosensory investigation of this syndrome, could open new connections between purely clinical aspects, such as the identification of a potential biomarker, and basic research aspects, to understand how and at what time a genetic alteration can modify a sensory and subsequently perceptive and / or cognitive response. A future study on 3M syndrome will be to carry out, through a longitudinal study, the evaluation of any further psychophysiological, behavioural and cognitive variations connected to olfactory aspects.

## Figures and Tables

**Table 1:** Results of descriptive analysis of amplitude (µV) and latency (ms) of N1 and Late Positive Component (LPC) in 18-month-old 3M-O and HS-O subjects.

	Group	N1 Amplitude	N1 Latency	LPC Amplitude	LPC Latency
Frontal Left	3M-O	−17.34	66	33.32	191
	HS-O	−1.42	137	10.05	328
Frontal Right	3M-O	−25.37	125	13.40	230
	HS-O	−12.55	234	3.85	188
Central Left	3M-O	−11.17	141	24.02	180
	HS-O	−4.10	160	12.9	402
Central Right	3M-O	−7.97	164	9.75	282
	HS-O	−2.70	102	12.23	203
Temporal Left	3M-O	−6.80	140	24.4	188
	HS-O	−2.20	105	18.28	227
Temporal Right	3M-O	−4.52	148	14.83	2,93
	HS-O	−14.5	113	8.83	2,54
Cz	3M-O	−4.28	109	1.51	328
	HS-O	−11.80	129	1.20	262
Fz	3M-O	−6.97	164	2.57	254
	HS-O	−28.24	133	9.38	191
Pz	3M-O	−2.80	160	4.26	246
	HS-O	−1.34	113	22.06	156

**Table 2:** Results of the descriptive analysis of the averaged amplitude (µV) and latency (ms) for N1 and LPC in 3M-N and HS-N. Two dashed lines indicate a lack of signal.

	Group	N1 Amplitude	N1 Latency	LPC Amplitude	LPC Latency
Frontal Left	3M-N	−3.23	121	6.56	267
	HS-N	−8.94	203	4.89	297
Frontal Right	3M-N	−9.32	188	--	--
	HS-N	−2.38	184	--	--
Central Left	3M-N	−7.68	121	5.88	262
	HS-N	−5.76	168	4.19	297
Central Right	3M-N	−1.47	195	1.30	215
	HS-N	−6.49	148	--	--
Temporal Left	3M-N	−5.85	105	0.311	297
	HS-N	−9.26	172	5.08	316
Temporal Right	3M-N	−0.988	137	6.07	207
	HS-N	−2.15	164	7.85	270
Cz	3M-N	−16.02	180	--	--
	HS-N	−11.80	129	1.20	262
Fz	3M-N	−8.95	109	5.78	160
	HS-N	−6.67	223	9.58	250
Pz	3M-N	−6.12	0,27	14.27	164
	HS-N	−9.96	105	5.58	188
					

**Table 3:** ROI Analytic evaluation.

ROI	3M-O/HS-O1	3M-O/HS-O2	3M-N1/HS-N1	3M-N1/HS-N2	3M-N2/HS-N1	3M-N2/HS-N2
Temporo-Parietal Right	57.82%	54.81%	10.78%	10.23%	23.25%	22.77%
Parietal	68.92%	45.61%	66.30%	74.88%	73.48%	80.24%
Temporo-Parietal Left	47.01%	54.58%	10.99%	29.73%	29.63%	44.44%
Central Right	19.48%	37.37%	−3.52%	46.62%	−79.17%	7.61%
Central	57.82%	89.46%	0.95%	−13.04%	25.93%	15.46%
Central Left	53.19%	67.60%	17.89%	28.60%	−20.00%	−4.35%
Frontal Right	40.38%	34.71%	33.33%	39.13%	35.90%	41.47%
Frontal	45.61%	76.69%	22.96%	−25.60%	69.70%	50.59%
Frontal Left	49.81%	58.67%	35.00%	27.78%	16.67%	7.41%

Percentage difference, by ROIs, between the green regions (area of the power spectrum ≤ 4 Hz) in Figure 3 for subjects with 3M syndrome and control subjects. Data that exhibit concordant positive values are highlighted in orange.
